# Self-growth suppression in *Bradyrhizobium diazoefficiens* is caused by a diffusible antagonist

**DOI:** 10.1093/ismeco/ycaf032

**Published:** 2025-02-17

**Authors:** Armaan Kaur Sandhu, Brady R Fischer, Senthil Subramanian, Adam D Hoppe, Volker S Brözel

**Affiliations:** Department of Biology and Microbiology, South Dakota State University, 1224 Medary Avenue, Brookings, SD 57007, United States; Department of Chemistry, Biochemistry and Physics, South Dakota State University, 1224 Medary Avenue, Brookings, SD 57007, United States; Department of Biology and Microbiology, South Dakota State University, 1224 Medary Avenue, Brookings, SD 57007, United States; Department of Chemistry, Biochemistry and Physics, South Dakota State University, 1224 Medary Avenue, Brookings, SD 57007, United States; Department of Biology and Microbiology, South Dakota State University, 1224 Medary Avenue, Brookings, SD 57007, United States; Department of Biochemistry, Genetics and Microbiology; Forestry and Agricultural Biotechnology Institute, University of Pretoria, Lunnon Road, Pretoria, South Africa

**Keywords:** *Bradyrhizobium*, antagonism, kin rivalry, sibling rivalry, self-growth suppression

## Abstract

Microbes in soil navigate interactions by recognizing kin, forming social groups, exhibiting antagonistic behavior, and engaging in competitive kin rivalry. Here, we investigated a novel phenomenon of self-growth suppression (sibling rivalry) observed in *Bradyrhizobium diazoefficiens* USDA 110. Swimming colonies of USDA 110 developed a distinct demarcation line and inter-colony zone when inoculated adjacent to each other. In addition to self, USDA 110 suppressed growth of other *Bradyrhizobium* strains and several other soil bacteria. We demonstrated that the phenomenon of sibling rivalry is due to growth suppression but not cell death. The cells in the inter-colony zone were culturable but had reduced respiratory activity, ATP levels, and motility. The observed growth suppression was due to the presence of a diffusible effector compound. This effector was labile, preventing extraction, and identification, but it is unlikely a protein or a strong acid or base. This counterintuitive phenomenon of self-growth suppression suggests a strategic adaptation for conserving energy and resources in competitive soil environments. *Bradyrhizobium’s* utilization of antagonism including self-growth suppression likely provides a competitive advantage for long-term success in soil ecosystems.

## Introduction

Microbes in soil encounter numerous challenges that influence their population dynamics and success. Sporadic or seasonal nutrient input and the generally high microbial densities pose a challenge for microbial survival. Soil microbial populations displaying long-term success are, therefore, expected to use these finite nutrient resources sparingly [1]. Nutrient availability determines growth yield, and low nutrient conditions select oligotrophs over copiotrophs [2]. Spatial constraints within microbial communities are an additional challenge, limiting physical space, growth, and dispersal [3]. Microorganisms express diverse social behaviors, and many can recognize their neighboring microbial populations for cooperation or competition [4]. Microbes also face challenges from surrounding microbial communities, and many microbial taxa have evolved mechanisms to suppress their competitors [5]. Some taxa use long-range strategies to inhibit others while others use contact-dependent or short-range mechanisms. One prominent long-range example is the production of antimicrobial compounds like antibiotics and bacteriocins. Antimicrobial diffusion through the soil inhibits the growth of nearby competitors who no longer consume resources, giving the antagonist a competitive advantage [6–9]. Other long-range mechanisms include iron-scavenging systems to deprive competitors, suppressing their growth [10]. Short range or contact dependent mechanisms include the type VI secretion system (T6SS), where syringe-like protrusions inject toxins into the competitors [6, 11–13].

Successful competitive strategies typically involve identification and differentiation between genetically related (self) and unrelated individuals (non-self) [14, 15]. Microbes have various ways of differentiating among self and non-self. The most widely researched is quorum sensing (QS), where population density is measured through extracellular concentration of autoinducer [16]. Some autoinducer systems are strain or species-specific, while others act across species. In biofilm co-cultures *Agrobacterium tumefaciens* biomass decreased when occurring with wild-type *Pseudomonas aeruginosa*, but remained constant with a mutant lacking QS abilities, indicating competitive interactions [17]. Another phenomenon to differentiate self and non-self is the toxin–antitoxin (TA) system, comprised of two elements: a stable toxin that inhibits a cellular process and an antitoxin of short half-life that counteracts the cognate toxin [18]. Many bacterial populations use TA systems for cell contact-dependent killing of competing species, including *Vibrio cholerae*, *P. aeruginosa*, and *Caulobacter crescentus* [12, 19, 20]. In *Myxococcus xanthus*, a model organism for exploring social behavior, swarm colonies of identical strains merge, while strains expressing different SitA toxin cassettes are able to discriminate, forming demarcation zones, termed kin rivalry [21]. While kin rivalry between nonidentical strains is well established, isogenic strains of swimming *Marinobacter algicola* and *Pseudomonas putida*, and swarming *Paenibacillus dendritiformis* develop a boundary between two colonies, termed sibling rivalry [22–25]. Swarming *P. dendritiformis* secrete subtilisin which promotes colony expansion. They also secrete a 20kD protein that is cleaved by subtilisin to form the 12 kDa toxin Slf. The concentration of subtilisin and the pre-toxin exceed the threshold at the interface between colonies, causing growth-inhibitory conditions [26]. *M. algicola* arrest in swimming populations has been linked to secreted glycerophosphoryl diester phosphodiesterase (GDPD), but its role in this process remains unclear [24]. No chemical compounds were detected in the case of *P. putida*, where compression waves among opposing colonies cause the development of the demarcation line [23]. Recently, Kastrat, and Cheng reported inhibition within and across *Escherichia coli* strains and other bacteria [25]. This broad-spectrum inhibition appeared dependent on a secreted inhibitory compound as it was independent of nutrient depletion and QS, but the nature of the compound was not reported. Sibling rivalry does not appear due to a conserved mechanisms, but in all but one case is shown to be due to a threshold concentration of toxin that accumulates at the interface between two swimming or swarming colonies.

We unexpectedly discovered sibling rivalry between two swimming isogenic colonies of *Bradyrhizobium diazoefficiens* USDA 110, a biological nitrogen fixer in soybean root nodules [27]. While studying competition among swimming *Bradyrhizobium* strains, two isogenic colonies of USDA110 used as the negative control also paused swimming upon approaching each other, forming a demarcation line. The phenomenon of suppressing of self is counter intuitive as *Bradyrhizobium* are slow growing, and to modulate, must compete in a rhizosphere teaming with faster-growing taxa supported by root exudates [28]. Here, we report (i) characteristics of this phenomenon and (ii) experiments to understand the underlying mechanism. This behavior of *B. diazoefficiens* is intriguing and unique because it includes restraining population growth of self, other *Bradyrhizobium*, and other soil bacteria. This phenomenon suggests a finely tuned strategy for limiting resource utilization as opposed to the concept of the copiotroph, the voracious species that eats as much as it can to grow its population as large as it can. These findings help explain how the slow-growing *Bradyrhizobium* is the most predominant bacterial genus in soils across the planet [29].

## Materials and methods

### Bacterial strains and culture conditions

*Bradyrhizobium* strains, mutants of *B. diazoefficiens* USDA110, and other bacteria used are listed in Table S1. USDA110 was genetically tagged with bjGFP, mTq2, sYFP2, and mChe following the originators’ method [30]. The plasmid pRJPaph-gfp, generously provided by Hans-Martin Fischer, was transformed into *E. coli* S17–1 λpir, and then subsequently transferred to USDA 110. USDA 110 was cultivated in PSY medium supplemented with L-(+)-arabinose (1 g/l) [31]. Cultures were incubated at 28°C and shaken at 250 rpm. The medium was supplemented with 0.35% agar for motility experiments and a 48 h exponential phase culture grown in PSY was used as an inoculum. We also used filter-sterilized soil extract (SESOM) as a motility medium to replicate the nutritional conditions found in soybean field soil [32, 33].

**Figure 1:**
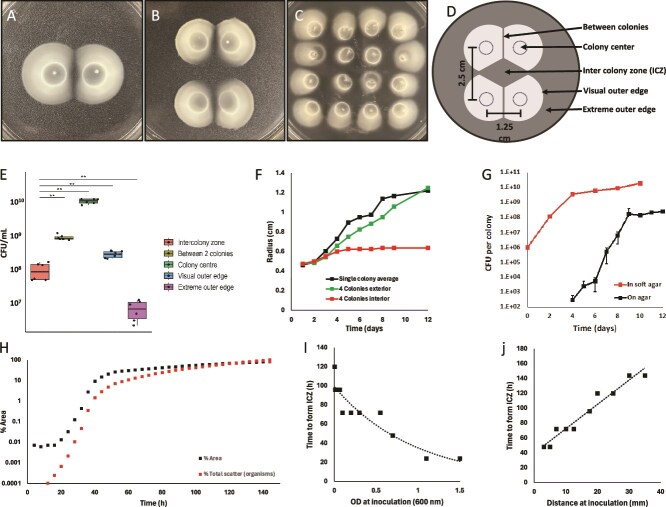
Sibling rivalry and colony arrest in *B diazoefficiens* USDA 110 are indicated by the formation of an ICZ between 2, 4, and 16 colonies on swim agar, demonstrating spatial patterns of colony arrest (A–C). The experimental setup used to investigate ICZ and colony interactions is shown as a schematic diagram (E). The number of culturable cells per zone, highlighting differences in cell viability and density within the ICZ compared to other areas (F); the culturable count of single colonies growing on 1.5% agar versus the total biomass of a 4-colony setup (G); colony expansion over time, illustrating dynamic changes in colony size and ICZ formation (D); the time needed to form the ICZ as a function of initial cell density (H); and the distance between colonies, providing insights into ICZ kinetics (I). Panel J shows the surface area of all colonies and the cell density as a function of light scatter in a 4-colony setup (J).

To visualize swimming, 20 μl of USDA 110 diluted to an absorbance (600 nm) of 0.100 was spotted onto motility agar and incubated for 10 days or as specified. Plates were inoculated with either 2, 4, or 16 droplets of the inoculum, (Fig. 1A–C). However, for most of the subsequent experiments, 4 droplets were used as standard. All other strains of *Bradyrhizobium* and 7 swimming soil bacterial species (Table S1) were inoculated in the same manner.

### Cell density quantification across colonies

To determine the density of culturable cells across the colony, we partitioned the colony into five specific locations: (i) inter-colony zone (ICZ), (ii) the area between two colonies, (iii) the colony center, (iv) the outer edge, and (v) the extreme outer edge (Fig. 1D). We collected 100 μl of agar from each of the five locations into separate tubes and 900 μl of sterile water was added, and the suspension was vortexed for 30 s, shaken for 10 min, and vortexed for 30 s. Colony forming unit/ ml (CFU/ml) was determined by plating 20 μl droplets of 10-fold dilutions in triplicate onto PSY agar and incubating for 5 days.

### Expansion visualization

To quantify colony expansion and time taken to develop the demarcation line, 4-colony setup plates were incubated for 12 days, and the radius of expanding inner and outer edge was measured every 24 h. Colony expansion was visualized using a scanner (Epson Perfection V370 Photo) to capture images every 4 h, (0 h to 140 h). Images were compiled using the montage feature of ImageJ software [34]. Scanner images were converted to 32-bit grayscale in Fiji [35]. The mean background was measured, image stacks were made for each colony and an intensity threshold was applied at the minimum detectable amount of cells corresponding to the ring at 4 h. The area occupied by the colonies was quantified by the number of pixels occupying the threshold and the number of normalized number of organisms was quantified by the intensity originating from light scatter within the threshold. Colonies started with absorbance from 0.005 to 1.5 were observed every 12 h to determine relationship between time taken to develop the demarcation line and concentration of the starter inoculum. To investigate the role of initial distance, 20 μl at the absorbance of 0.100 nm was spotted from 3 mm to 35 mm apart and observed every 12 h.

### Growth inhibitory effect on other strains and species

To determine whether USDA 110 impacted the growth of other *Bradyrhizobium* strains and other bacterial species (Table S1), a 10 μl drop of the specific strain (absorbance of 0.100) was introduced onto the ICZ of a 10-day-old colony with specific control. To rule out nutrient depletion, experiments were repeated using PSY with five-fold arabinose, and also overlaid after 10 days with fresh soft PSY before spot-inoculating *B. subtilis* and *P. aeruginosa*.

### Space maintenance

To determine cell distribution within swimming colonies, bjGFP tagged USDA 110 inoculated onto motility agar in glass-bottom MatTek dishes (35 mm petri dish, no. 1.5 cover glass, P35G-1.5-14-C). Widefield images ICZ boundary, colony center, and outer edge were captured using an inverted microscope built around a Till iMic (Till Photonics, Munich, Germany) equipped with a 60X 1.2 N.A. water-immersion objective. Imaging was done from 237 to 321 μm with step size of 2 μm at excitation/emission of 488/510 nm and 560/618 nm for bjGFP and mChe respectively. Z stack was captured for each fluorophore and the resulting image sequence was processed in FIJI, with images from depths of 245, 271, and 299 μm shortlisted [35]. Background was subtracted using the rolling ball background subtraction method (25-pixel radius), and the noise was reduced using a despeckle median filter.

### Physiological conditions of the cells

To determine whether colony arrest was due to death of cells, we stained colonies by flooding plates with BacLight (Molecular Probes; Kit L13152) [24], and viewed fluorescence after 15 min using a stereomicroscope (Olympus SZX16). Images from GFP and RFP filters were composited in ImageJ. For assessing cell respiratory activity across the colony, 0.01% 2,3,5-triphenyltetrazolium chloride (TTC, MP Biomedicals SR00767) was poured over 10-day-old colonies [24]. After 5 h at 25°C, plates were observed and imaged using iPhone 14. ATP content was measured using the BacTiter-Glo™ Microbial Cell Viability Assay Kit (Promega, Cat.G8230/2). Briefly, agar (500 μl) from five locations was mixed with 750 μl of P/10 (1/10 the peptone and yeast extract in PSY, without sugar [36]) and the CFU determined. The suspension was mixed by vortexing for 30 s, shaking for 10 min, and vortexing for 30 s. Suspension was centrifuged (30 000 *x g,* 20 min, 4°C), the pellet was suspended in P/10 to a total volume of 500 μl, 250 μl of ice-cold 1.2 M perchloric acid was added, the tubes were vortexed and incubated on ice for 15 min. After centrifugation (30 000 *x g*, 7 min, 4°C), 500 μl of the supernatant was neutralized with 250 μl of a mixture of KOH (0.72 M) and KHCO_3_ (0.16 M). Following centrifugation, 100 μl of the supernatant was transferred to a well of a 96-well glass plate, 100 μl of Bac Titer-Glo reagent was added, and shaken for 5 min in the dark. The reaction mixture was transferred to a 96-well white plate, and luminescence was read using a luminometer (BioTek Synergy2 Microplate reader). Relative luminescence units were normalized by CFU/ml obtained from each sample location.

We wanted to assess the movement of cells in the ICZ, colony center, and extreme outer edge of the swimming colony. Agar from each location was pooled to 7 ml, resuspended in 3.5 ml of sterile P/10 medium, vortexed for 30 s, shaken for 10 min, and vortexed again for 30 s. Then, 3 ml was added to three wells of a 12-well plate, and the remaining sample was used to determine the initial CFU. Three glass capillaries filled with PSY arabinose (chemoattractant) were placed in each well, allowing cells to migrate toward the attractant >1 h [36]. After 60 min, the capillaries were emptied into microcentrifuge tubes, and the CFU of motile cells determined. The proportion of moving toward the attractant was calculated using initial and motile cell CFU. To gain insights into the role of the two known flagellar systems of USDA 110, colony expansion in a 4-colony-setup of WT was compared to the delta-*fliC* (lacking subpolar flagella), the delta-*lafA* (lacking lateral flagella), and delta-*fliC lafA* strains [37].

### Possible causes of arrest in colony expansion

To investigate whether nutrient depletion caused colony arrest, we supplemented the ICZ and the area between two colonies with 30 μl of 10-fold PSY medium on the 4th and 7th day of the 10-day incubation. Plates were observed daily, and images were recorded to monitor colony expansion changes. Simultaneously, we reduced agar density to 0.15% to accelerate colony migration and shorten the time for colonies to meet, minimizing nutrient consumption. To visualize a possible role of compression waves in ICZ development [23], we positioned a sterile glass slide vertically between two swimming colonies and observed daily for demarcation line development. In a parallel experiment, colonies inoculated near the Petri plate edge were monitored for expansion and demarcation line formation. Compression waves from the colonies would interact with the glass slide or plate edge, potentially halting cell movement at the colony boundary.

To investigate whether colony arrest was due to a diffusible compound, we used a permeable membrane that allowed diffusion but not cell passage. We placed an autoclaved 0.2 μm pore size filter membrane (Isopore PC Membrane, LOT:0000283982) over 10-day-old bjGFP single and a set of two swimming colonies. ICZ extracted from five plates was placed onto fresh motility plates, overlaid with membrane. This procedure was repeated using autoclaved and UV-treated ICZ. We poured 2 ml of soft agar onto the center of the filter membrane, and after solidifying, spot-inoculated with mChe expressing liquid inoculum (10 μl) at an absorbance of ~0.100. The plates were observed for growth and motility of second drop inoculation (mChe) and imaged after 7 days. To evaluate the effect of lower colony on growth above in absence of potential diffusion, we used membrane that allows only water to pass (Dow Filmtec Flat Sheet Membrane, SW30XFR, PA-TFC). As an additional confirmation for the presence of a diffusible compound, transwells with 0.4 μm pore size membrane (Corning Costar 3450-clear) were placed over a 10-day-old 4-colony setup and a sterile plate. Fresh liquid culture (300 μl, absorbance set at 0.035 at 600 nm) was added to the transwell, incubated for 48 h when CFU/ml was determined. To investigate role of QS and isovaleryl-homoserine lactone (IV HSL), the *bjaI* mutant (AL17) [38] was inoculated as a 4-colony setup, and colony expansion was observed.

### Nature of the effector

To extract the potential effector, the agar between 4 colonies “ICZ region” was collected from 40 plates and pooled into 50 ml tubes yielding 15 ml. Four such tubes were prepared for extraction. The 15 ml extracted ICZ was suspended in 30 ml of either ethyl acetate, methanol, or water, vortexed for 1 min and ultrasonicated with 5 pulses of 1 min each with intermittent cooling on ice for 30 s. Tubes were centrifuged at 7000 *x g* for 15 min, and the supernatant was aliquoted into 2 ml tubes and concentrated in a speed vac for 3–4 h. Once dry, the tubes were refilled with 2 ml extract twice to increase the quantity of concentrate. The concentrated extract was stored at 4°C until used. The extracts were resuspended in 100 μl of water, but the extract from ethyl acetate was suspended in 10% ethyl acetate. Suspensions were applied as 5 μl droplets at the edges of a 4-day-old swimming colony, and the plates were observed for a halt in colony expansion. To assess the extracts’ impact on growth rate of USDA 110, 20 μl of extract was added to 150 μl of fresh culture, with nine replicates inoculated in 96-well plates. Growth was monitored in a plate reader (FLUOstar Omega reader, BMG LABTECH) for 36 h at 600 nm at 28°C. To detect protein, 30 ml of pooled ICZ was suspended with 15 ml of sterile water by vortexing and shaking steps [24]. Briefly, samples were frozen at −80°C for 2 h, thawed, and centrifuged (30 000 *x g* at 4°C for 20 min) to remove pellets containing cells and debris. The supernatant was subjected to TCA and 80% ammonium sulfate precipitation. The precipitate was centrifuged, suspended in PBS, and placed in a 3 kDa spin column (Thermo Scientific, 88 512), and centrifuging at 30000 *x g* for 10 min. The concentrate was resolved in a 12% SDS PAGE gel and stained with Coomassie Brilliant Blue R250, destained, and viewed. We also employed SYPRO Orange Protein stain (Thermo Fisher S6651) on the ICZ.

To assay for possible volatile compounds produced by *Bradyrhizobium* in the soft agar, we placed transwells with 0.4 μm pore size membrane over 10-day-old 4-colony setups and added 1 mL sterile Milli Q water. Transwells over uninoculated soft agar served as negative control. After 24 h, water was withdrawn and analyzed using an Agilent 8890/7000E GC/TQ with PAL RTC 120 autosampler. The software for data acquisition and analysis was *Enhanced MassHunter*, with library *NIST23*.

### Statistical analysis

Experiments were conducted in triplicate biological replicates, each with three technical replicates. Fisher’s LSD test were performed in R to determine statistically significant differences between the means of different groups [39]. Asterisks denote levels of significance in *P*-values: *P* < .05: ^*^; *P* < .01: ^*^^*^; *P* < .001: ^*^^*^^*^. R packages used included dplyr, agricolae, ggplot2, ggpubr, scales, readxl, and patchwork.

## Results

### Characterization of the biological phenomenon

The development of a demarcation line between encroaching swimming or swarming colonies is indicative of spatial inhibition and has been observed between different strains of a species [40]. However, sibling rivalry (competition between isogenic colonies) is rarely documented and studied. Here we observed identical *Bradyrhizobium* strains develop a demarcation line when swimming colonies are inoculated nearby, indicating that each colony prevents the expansion of the other (Fig. 1A). This behavior was observed in five motile strains evaluated: *B. diazoefficiens* USDA 110 (Fig. 1A), *B. elkanii* USDA 83, *B. japonicum* USDA 20, *B. arachidis* USDA 3384, and *B. liaoningense* USDA 13 (Fig. S1). *B. diazoefficiens* USDA 110 was chosen for characterizing the phenomenon due to its agricultural significance in nitrogen fixation. The observed behavior is not specific to the growth medium, occurring both in PSY-arabinose and SESOM soft agar. Four colonies had a more pronounced inhibition zone, which we termed “inter-colony-zone” (ICZ; Fig. 1D). Colonies at the center of a 4 × 4 grid were suppressed the most, and the outer-most colonies showed expansion at their exterior edge and colony arrest at inner edges facing other colonies (Fig. 1C). The inhibition zone became more distinct with an increase in the number of inoculation points, indicating that the expansion arrest is related to regional cell density. Most experiments were the 4 colony setup (Fig. 1E). Colonies of other soil bacteria able to swim in 0.35% agar did not display colony expansion arrest (Fig. S2). To see if cells were evenly distributed across colonies, we determined the culturable count at five different locations of the 4-colony system (Fig. 1E). The colony center contained the highest cell density, followed by the area between the two colonies (demarcation line) and the visual outer edge. Although the agar in regions outside of colonies appeared clear, ~10^7^ CFU/ml were detected 4 mm away from the visible colony boundary, suggesting that a subset of the colony population swam ahead like scout cells (Fig. 1E). Similarly, the occurrence of ~10^8^ CFU/ml in the ICZ suggested that a subset of the colony population swimming inward was suppressed and did not attain full density after one month of incubation. The presence of cells in the extreme outer edge and the ICZ was confirmed by fluorescence microscopy of GFP-tagged cells.

To characterize colony expansion arrest, we tracked in- and outward facing radii of the 4-colony setup versus single colony. Until day 4, colonies expanded in all directions, but then inward expansion stopped while outward expansion continued at the same rate as a single colony (Figs 1F and S3). To determine whether growth arrest is unique to swimming colonies, we compared growth rates of cells in 4-colony setups (0.35%) versus colonies developing from single cells on solid agar (1.5%) through CFU counting. Colonies developing from single cells became visible after 4 days, with a generation time of 6.3 h until day 9, then slowing to 123 h, essentially stationary phase (Fig. 1G). However, the generation time of cells in swim colonies was 8.1 h during the first 4 days, slowing to 59.5 h from day 4. The slower generation time in soft agar indicated some growth inhibition from the start. We also confirmed growth suppression by quantifying cell biomass of scanned colonies. After 40 h colony expansion rate decreased (Figs 1H and S3). The rate of increase of biomass also decreased after 40 h, as reflected by the pixel number per colony. The discrepancy between onset of slower growth rate (Fig. 1G) and biomass increase (Fig. 1H) may be because the cells became smaller between 40 and 96 h.

**Figure 2:**
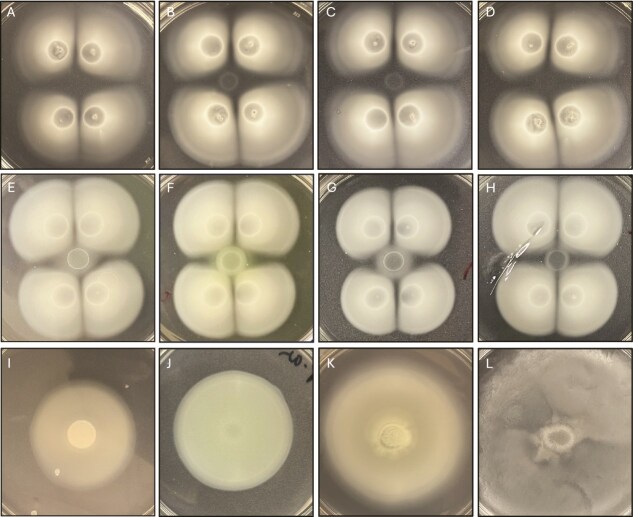
Inhibition of growth in the ICZ of swimming colonies of *B. diazoefficiens* USDA 110. Panels A–D were spot-inoculated onto the ICZ with USDA 110, USDA 20, USDA 140, and USDA 83. Panels E–H were spot-inoculated onto the ICZ with *Streptomyces ATCC*, *P. aeruginosa PA01*, *Herbaspirillum seropedicae*, and *B. subtilis 168*, while panels I–L contained only the respective single cultures.

The initial cell number inoculated for founder colonies affected the time taken to ICZ (Fig. 1I). This indicated that demarcation lines form irrespective of initial inoculum density, with lower inocula requiring more time to reach the cell density needed for colony arrest. Distance between founder colonies appeared linearly related to ICZ development time (Fig. 1J). The greater the initial distance, the more time was required for expanding colonies to reach proximity. Overall, ICZ formation was a function of cell density and colony proximity. To determine whether reduced cell density in the ICZ was due to growth suppression, we introduced a fresh inoculum of USDA 110 to the ICZ of a 10-day-old 4-colony setup. Even after 10 days of incubation, no visible growth was observed (Fig. 2A), suggesting a growth-suppressing phenomenon associated with swimming colonies of USDA 110. To determine cell distribution of USDA 110 in the swimming colonies, we performed confocal microscopy on bjGFP tagged USDA 110, and individually pre-cultured mChe, bjGFP, sYFP2, and mTq2 populations mixed before inoculating. While bacteria are widely known to form multicellular aggregates, both on surfaces and in swim agar [41, 42], USDA110 maintained spaces among cells (Fig. S4A–C), distinct from *E. coli* (Fig. S5A). Segregation was also observed in a mixed culture of mChe and bjGFP on hard agar (Fig. S5B).

### Growth inhibitory effect on other strains and species

We asked whether the accumulated growth suppressing substance in the ICZ also affected other soil bacteria. To evaluate this we added cell suspensions of *Bradyrhizobium* and other soil bacterial species to 10-day-old 4-colony setups. All *Bradyrhizobium* strains were inhibited, but USDA 26, 140, and 83 exhibited limited growth within the ICZ (Fig. 2B–D). Growth and motility of *Streptomyces* ATCC 49182, *P. aeruginosa* PA0, *Herbaspirilum seropedicae* ATCC 33892, and *B. subtilis* 168 were suppressed when compared to swimming on their own (Fig. 2E–L), as were *Arthrobacter aurescens* TCI, *E. coli* K 12, *Pseudomonas* ADP, and *Salmonella Typhimurium* (Fig. S6). To exclude nutrient depletion prior to inoculating of other species, we cultured USDA110 in PSY with five-fold arabinose, and also overlaid this with fresh PSY soft agar prior to inoculating *B. subtilis* or *P. aeruginosa*. Neither mode of nutrient supplementation alleviated suppression of these (Fig. S7). These data indicate that the growth-suppressing phenomenon affects not only USDA 110 but also other strains of *Bradyrhizobium* and other fast-growing species of soil bacteria.

### Physiological state of the cells in the inter-colony zone

**Figure 3:**
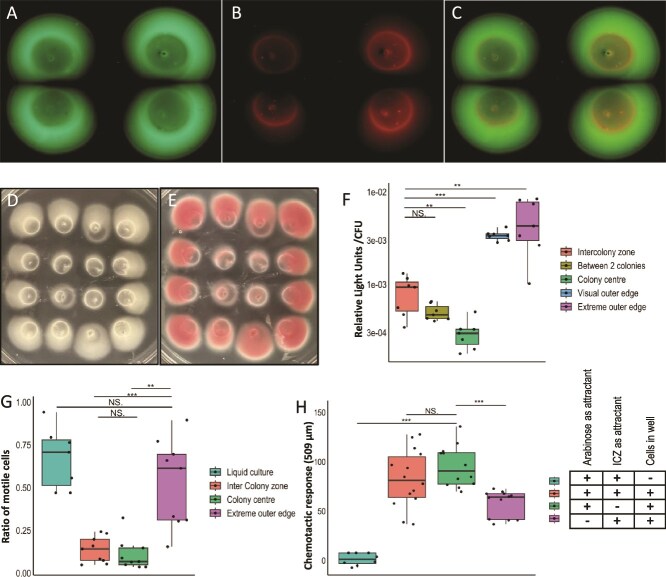
Physiological state of cells across swimming colonies of USDA 110. Panels A–C show four colonies stained with SYTO 9 and propidium iodide and merged to differentiate live and dead cells. Panels D and E show a 16 colony setup before and after staining with TTC, which turns pink through reduction linked to electron transport, indicating respiratory activity. Panel F shows ATP per cell in the five locations. Panel G shows the motility of cells taken from different locations toward arabinose compared to liquid culture. Panel H shows the chemotactic response of USDA 110 to ICZ, arabinose or both, as quantified by fluorescence.

The different growth rates and cell densities pointed to physiological differences among populations in the various zones. We assessed the physiological state of the cells across colonies and in the ICZ. BacLight was used to visualize live versus dead cells, TTC reduction was used to visualize respiratory activity, and cellular ATP content was quantified. Baclight stained colonies fluoresced green throughout (Fig. 3A). Only the circumference of the initial area of inoculation fluoresced red, indicating dead cells at the origin of the colony (Fig. 3B and C). No red fluorescence was observed at the colony edge, even the part facing the ICZ, indicating that expansion arrest was not due to cell death. This was further supported by the culturability of cells from the ICZ (Fig. 1E). Furthermore, the transfer of sections of the ICZ onto sterile agar results in visible growth after incubation. Populations closer to the ICZ and colony edge displayed less TTC reduction than at the colony center (Fig. 3D and E). Colorless TTC is reduced to pink 1,3,5-triphenylformazan by succinate dehydrogenase, a function of respiratory activity [43]. This indicated that cells between colonies and at the outer edge showed lower respiration activity than at the colony center. The greatest suppression was observed in the 4 colonies situated in the center of the 4 × 4 grid, marked by minimal expansion and a light pink color with white-tinted areas. ATP levels varied significantly across colonies (Fig. 3F). Cells in the colony center had the least ATP, followed by those between the colonies and the ICZ. Cells at the outer and extreme outer edges had the most intracellular ATP. Cells at the colony center displayed high respiratory activity but low ATP, indicating high energy demands, cells between colonies had lower respiration and ATP, indicating lower activity but high energy demands. Conversely, outer edge cells had lower respiration but high ATP, suggesting lower demands on cells.

To determine cell motility across the colony, we quantified swimming toward the attractant arabinose. While outer edge populations displayed motility as cells grown in liquid culture, populations in the ICZ and the colony center displayed significantly lower swimming (Fig. 3G). This indicated that the observed phenomenon of colony arrest is also associated with a lack of motility. Because USDA 110 expresses at least two flagellar systems [44], we evaluated colony expansion by mutants deleted for *lafA, fliC*, or both. The single knockouts showed colony expansion and arrest like wild type, indicating that each flagellar system independently supports expansion (Fig. S8). As sibling rivalry was observed in both single flagellar mutants, the inhibitory function is not linked to either system. The double mutant did not swim.

To determine if the ICZ acts as a chemorepellent, we compared swimming toward ICZ agar and the known attractant, L-(+)-arabinose. There was no significant difference in motility between swimming toward the two, indicating that the ICZ did not exhibit chemo-repellent properties (Fig. 3H). These results indicate that cells in the ICZ lose motility and respiratory activity but are not chemotactically repelled and remain viable.

### Possible causative agent of colony arrest and growth suppression

Suppression of growth, ATP, and motility in the ICZ pointed to challenges to the cells. To explore possible causes of expansion arrest and growth suppression, we explored hypotheses regarding nutrient depletion, compression waves, and diffusible compounds or effectors. Assuming that decreased incubation time would lead to decreased nutrient consumption, we decreased agar density to 0.15% to decrease time required for expansion. Reducing agar density to 0.15% accelerated colony expansion, causing arrest and ICZ formation after 2 days instead of 4. Addition of droplets of 10 times higher concentration of nutrient to the ICZ 4 and 7 days after inoculation had no noticeable impact on colony arrest (Fig. S9). Hence, nutrient depletion was ruled out as a cause. Espeso *et al.* proposed that the arrest of swimming populations of *P. putida* was due to the generation of compression waves that propagate through the medium toward the other colony [23]. We examined compression waves as a potential factor by introducing a glass slide as a physical barrier between two expanding colonies, and inoculated colonies close to the edge of the petri plate. Compression waves would reach these barriers, resulting in the formation of a demarcation line. Colony edges advanced to the glass and plastic boundaries without a demarcation line (Fig. S10), dismissing compression waves as a factor in *Bradyrhizobium* colony expansion arrest.

**Figure 4:**
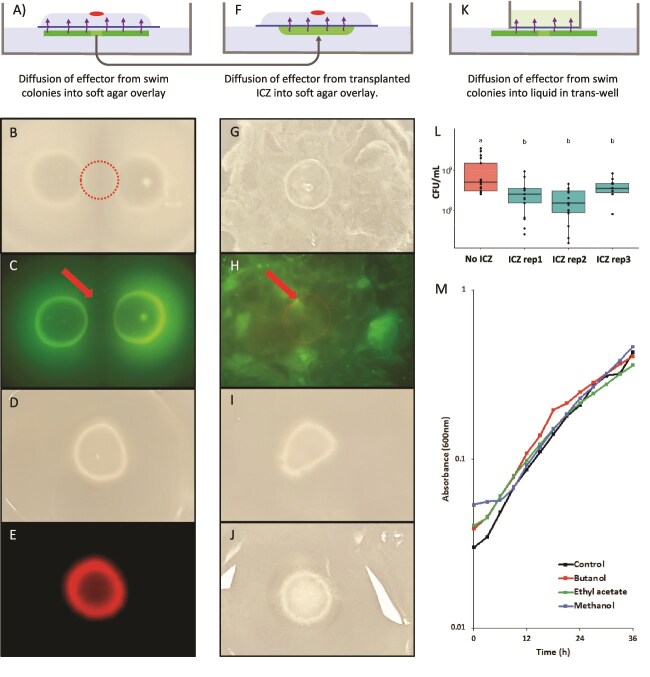
Experiments aimed at characterizing the cause of growth inhibition. Panels A, F, and K illustrate the three experimental setups, with a line representing a membrane allowing the passage of molecules. Panels B–C show mChe-tagged USDA 110 introduced to soft agar over a 10-day-old 2 colony setup as outlined in panel A. Panels D–E show mChe USDA 110 culture introduced to soft agar over a plate with no underlying culture. Panels G and H show mChe USDA 110 culture introduced to soft agar over a layer of ICZ and transferred onto sterile soft agar, as outlined in panel F. Panel I shows USDA 110 introduced to soft agar over a layer of UV-treated ICZ, while panel J shows overlay onto autoclaved ICZ. Panel L shows the culturable count of USDA 110 in liquid medium in a transwell plate (0.4 μm pore size) incubated on four 10-day-old colony plates versus noninoculated plates. Panel M shows growth in liquid supplemented with extracts from ICZ using different solvents.

To ascertain the possibility of a diffusible effector causing colony arrest, we employed 0.2 μm membrane filters to allow passage of diffusible compounds but not cells. Filters were placed onto 10-day-old two or single colony setups of bjGFP-tagged cells, overlaid with fresh motility agar, and spot inoculated with mCherry-tagged cells to observe growth suppression (Fig. 4A). Notably, the fresh inoculum on the top layer of agar did not develop into a visible colony, and no red fluorescence was detected (Fig. 4B and C). Similar observations were made with a single colony under the membrane. Growth above a membrane allowing only water to pass was uninhibited, suggesting that growth inhibition requires a diffusible compound. The inoculum placed on top of a system, with no swimming colonies at the lower layer of agar (sterile), grew into visible colonies and fluoresced red (Fig. 4D and E). These results indicated growth suppression, supporting the presence of a growth-suppressing diffusible compound produced by the underlying swimming colonies. To evaluate the presence of diffusible compounds specifically in the ICZ, we removed ICZ from 10-day-old 4-colony setups and transferred it either untreated, UV treated, or autoclaved onto fresh motility agar, followed by membrane overlays (Fig. 4F). Newly inoculated cultures grew only minimally, indicating the presence of diffusible compounds in the ICZ (Fig. 4G and H). During incubation of untreated ICZ, visible growth appeared as seen by green fluorescence, attributed to outgrowth of low number of cells present in the ICZ (Fig. 1F). To exclude contributions from this new cell activity, cells in ICZ were killed by either UV or heat treatment. New inoculum exhibited similar growth on both, as compared to the control without underlying ICZ (Fig. 4I and J). The lack of strong growth suppressing the impact of ICZ indicates that the effector had a short half-life or was sensitive to UV radiation and high temperature. Growth suppression over established colonies but far less over freshly outgrowing culture from ICZ indicates that the functioning of the effector is most active in the presence of live cells but has the ability to diffuse through agar and work at a distance. To assess growth inhibition by the effector in a liquid culture, we placed a 0.4 μm pore size transwell on 10-day-old colonies (Fig. 4K). Significantly lower yields were observed in transwells over swimming colonies than over sterile agar, indicating growth suppression (Fig. 4L). This reinforces the conclusion that a diffusible water soluble “Effector” produced by USDA 110 swimming colonies suppresses new culture growth.

With evidence for a diffusible effector, we sought to identify the nature of the compound responsible. We did not find a change in pH in the ICZ, indicating the absence of a strong organic acid or base as an effector. To test role of QS, we compared colony expansion of the wild type versus a *bjaI* deletion mutant shown to be deficient in QS [38]. As QS mutants behaved like the wild type, USDA 110’s known QS system does not cause colony arrest. Attempts to extract a possible protein from the ICZ by TCA or ammonium sulfate precipitation and separately by concentrating using 3 kDa cut-off spin filters showed no protein bands on SDS-PAGE. No fluorescence was detected after adding the protein-detecting SYPRO Orange Protein stain (Millipore Sigma). These results show that the effector is likely not a protein larger than 10 kD, but if it is, it has a short half-life. Unable to directly determine the nature of the effector, we performed extractions from ICZ using water, butanol, ethyl acetate, and methanol. Applying concentrated extract (5 μl) at various distances surrounding young colonies did not lead to visible arrest of expansion (data not shown). Likewise, the growth of liquid cultures supplemented with concentrated extracts grew at the same rate as that of untreated cultures (Fig. 4M). These observations suggest that the effector has a low half-life or is volatile and is lost in the extraction or concentration process.

To determine whether *Bradyrhizobium* in the soft agar produced water soluble volatile compounds, we placed 1 mL sterile MilliQ water into transwells on 10-day-old 4-colony setups. Growth inhibition in transwells over swimming colonies (Fig. 4L) had pointed to release of a diffusible effector into the aqueous environment. GC–MS analysis of water over *Bradyrhizobium* showed 32 peaks not found over sterile agar (Fig. S11). Conversely, several sterile agar peaks were no seen over *Bradyrhizobium*. None of the peaks matched any signature in the *NIST*23 library available with the GC–MS. Growth-suppressing activity may be due to only one or a combination from among these volatile compounds.

## Discussion

We report self-growth suppression in *B. diazoefficiens* USDA 110, initially observed as suppression of colony expansion, but exhibiting suppression of cellular activity and motility by surrounding cells through sibling rivalry. While sibling rivalry has been previously reported in *M. algicola*, *P. putida*, and *P. dendritiformis* [22–24], *Bradyrhizobium* is unique as it suppresses growth of itself (sibling rivalry), related strains (kin rivalry), and a broad cross-section of other bacterial taxa (antagonism). We characterized this phenomenon and report experiments to understand the underlying mechanism. Sibling rivalry is not unique to USDA 110, occurring across various *Bradyrhizobium* strains. The phenomenon also occurred in SESOM, an extract from soybean field soil, indicating occurrence in natural soil environments, thereby emphasizing the ecological relevance of expansion suppression beyond laboratory nutrition conditions.

Expansion suppression of *Bradyrhizobium* appears due to inhibition of cells rather than cell death. The generation time of cultures in soft agar was significantly slower than on hard agar (8.1 versus 6.3 h), indicating the onset of growth suppression from the beginning. The increase in population was associated with further slowing of average generation time in soft agar (~59 h from 4th day versus 6.3 h till day 9 on hard agar). Cell movement and growth cease in *Myxococcus xanthus* at low nutrient levels [40]. The shift to the quasi-stationary phase in USDA 110 occurred despite the availability of nutrients and space between cells, indicating a different basis for inter-colony rivalry. The onset of stasis was associated with an increase in population and time of populations occurring in agar. Cells in the colonies had reduced ATP/cell as compared to those at the colony outer edge. Furthermore, colonies surrounded by others displayed a reduced respiration rate (TTC reduction). Thus, cells surrounded by other colonies display lower overall activity and fitness than cells facing unpopulated space. Likewise, cells in colonies and the ICZ displayed low motility as compared to cells facing unpopulated space. The cells in freshly populated space displayed higher activity due to distance from established populations. A similar decrease in motility and respiration was observed during the study of sibling rivalry in *M. algicola* [24]. Bacteria, in general, spread outward for constant supply of a better nutritional environment [45]. MDR *Salmonella enterica* serovar Typhimurium has reduced motility, both swimming and swarming, in the presence of antibiotics [46]. While sibling rivalry in *P. dendritiformis* was caused by the death of cells [22], USDA 110 populations at the rivalry phase were culturable and stained live by BacLight. These observations point to suppression rather than killing as the cause of colony expansion suppression. However, the suppressive effect was strong enough to inhibit the growth of fresh inoculum on established ICZ (Figs 2A and  4B, C, and L).

A subset of cells was found outside of the visible colony boundary, both facing other colonies (ICZ) and unpopulated space. While cells in the ICZ were metabolically suppressed and did not display swimming, cells at the outer edge swam actively. Some of these cells behaved like scouts as they occurred far from the colony. This indicates a subset of the population displaying phenotypic heterogeneity. We have previously observed phenotypic heterogeneity in *B. diazoefficiens* USDA 110 while studying its surface properties [33]. Intriguingly, they did not grow locally to high density, indicating some restraint on cell division despite their ATP status. This swimming away from the colony could not be ascribed to chemorepulsion (Fig. 3H). The physiology of this subpopulation should be further studied.

Growth suppression by *Bradyrhizobium* appeared to be due to a diffusible compound with a short half-life. This effector of growth suppression was able to diffuse through the aqueous agar matrix and through pores in polycarbonate and polyester membranes. It lost activity after exposure to UV and heat, and during extraction and concentration procedures. Sibling rivalry in *P. dendritiformis* is caused by a two-protein system of the pre-toxin Slf and protease subtilisin [26]. *M. algicola* sibling rivalry was associated with GDPD, with glycerophosphoryl diester phosphodiesterase as the predicted effector [24]. The USDA 110 effector is unlikely to be proteinaceous as we were unable to obtain an extracellular protein from the ICZ. The effector is also not a strong acid or base, as no shift in pH was observed. It does, however, accumulate in soft agar over time and as a function of population size since colonies surrounded by multiple others were most suppressed (Fig. 3D and E). This behavior seems to be similar to the concentration-dependent mechanism of antibiotics [47]. The effector is clearly broad range, as it suppresses the growth of producers, members of the same genus, and a broad cross-section of other bacteria (Figs 2 and S6). Chemical characterization requires an extract with confirmed activity. Transmission of growth-suppressing action through pores in membranes indicates the effector is water soluble. Extracts obtained using solvents did not show growth-suppressing activity. This loss of growth-suppressing activity following extraction points to either a short half-life, or volatility. The detection of 32 GC–MS peaks associated with presence of *Bradyrhizobium* in soft agar points to production of multiple volatile compounds. Unfortunately, none of these compounds were identified using library provided with the instrument. Furthermore, pinning down which one or combination of these volatile compounds is associated with the phenomenon will require extensive future work.

Self-growth suppression or sibling rivalry leads to curtailing of the population, which should limit the success of the species in soil. *Bradyrhizobium* are slow growing and have to compete with diverse faster growing bacteria in soil and the rhizosphere [48]. These self-suppressing traits are counterintuitive in terms of long-term persistence and are expected to cause the species to be unsuccessful and therefore rare. Intriguingly, *Bradyrhizobium* is the most predominant genus found in soils globally [29], indicating success of the species in soil. This indicates that self-growth suppression, as described here, is part of the success strategy used by *Bradyrhizobium*. Copiotrophs such as *Bacillus* and *Pseudomonas* devour resources to maximize their population, outcompeting slower growing species [49–51]. Because nutrient input into soil is sporadic or seasonal, copiotrophs devour these limited nutrients, causing the community to starve [52, 53]. The ability of *Bradyrhizobium* to restrain or suppress growth and activity of itself and copiotrophs should reduce nutrient consumption, causing longer-term nutrient availability. This would enable the species to have longer-term success. We hypothesize that the self-suppressing mechanism indicates a strategic adaptation for conserving energy and resources in a competitive and nutrient-limited soil environment. This concept is similar to entering a dormant state and using fewer resources, giving it an edge over copiotrophs, especially in oligotrophic soil environments [54]. A similar strategy has previously been reported for bacteria growing slowly or becoming dormant under starvation conditions in soil as an alternative survival strategy [55]. By suppressing incoming bacteria, an individual *Bradyrhizobium* strain would dominate its location, facilitating its colonization of spaces such as root surfaces. This would benefit colonization, including nodulation of legume hosts [56]. We relate self-growth suppression to the long-term survival and competition strategy of this agriculturally beneficial genus.

## Supplementary Material

Supplementary_figures_ycaf032

Table_S1_ycaf032

## Data Availability

All the data generated for analysis during this study are included in this published article and its supplementary information files.
