# Supplementary material for: Self-growth suppression in Bradyrhizobium diazoefficiens is caused by a diffusible antagonist
Source: ISME Commun. 2025 Feb 17;5(1):ycaf032. doi: 10.1093/ismeco/ycaf032 (PMC11896636; doi:10.1093/ismeco/ycaf032)
Supplement: Supplementary_figures_ycaf032 [file supplementary_figures_ycaf032.pdf]

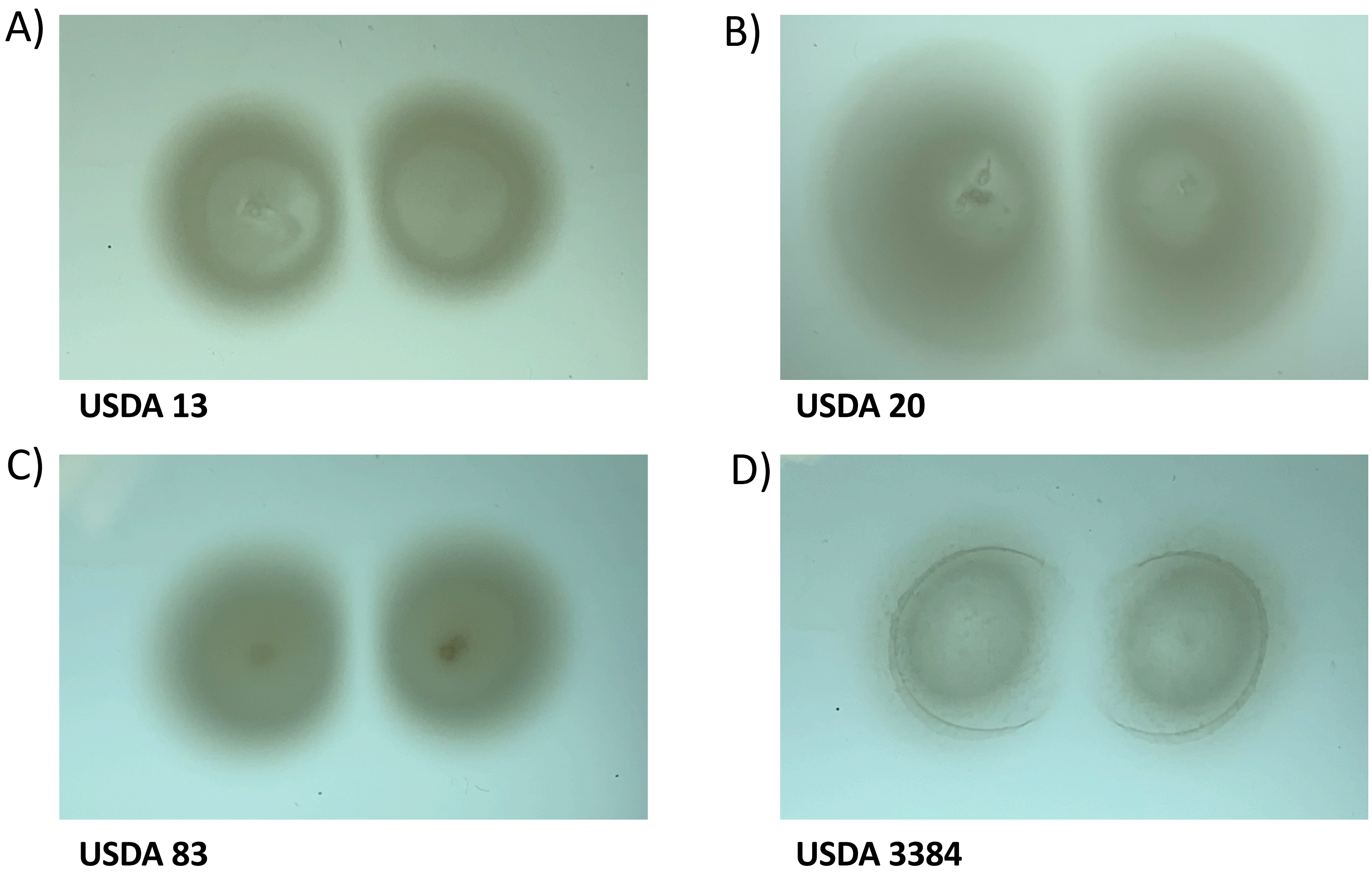

Figure S1 Two colony set-up for *Bradyrhizobium* USDA 13 (A), 20 (B), 83 (C) and 3384 (D) in PSY soft agar with arabinose, incubated for 7 d.

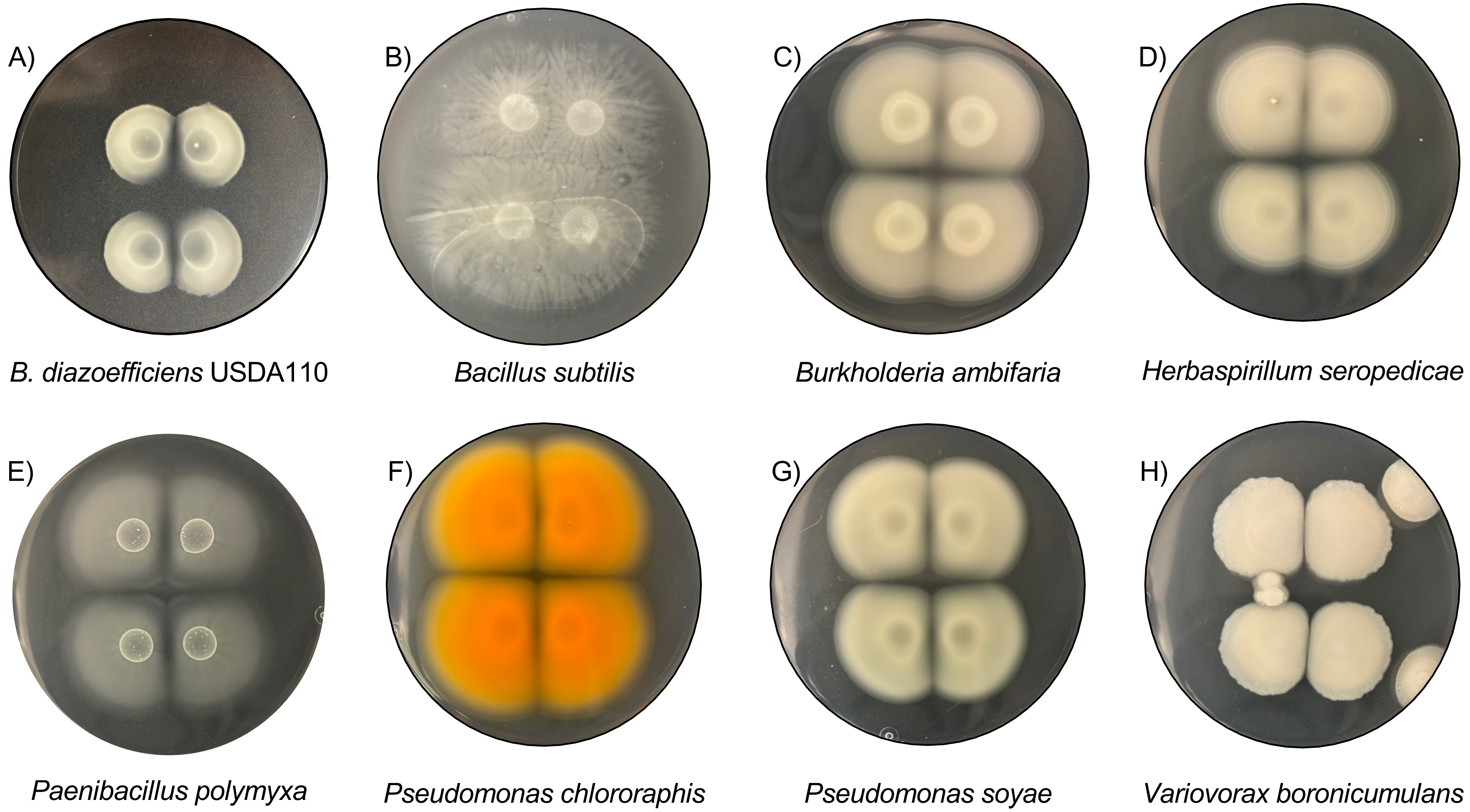

Figure S2. Four-colony setup of swimming soil bacteria showing either no or minor inter-colony zones when compared to *USDA110*. *Bradyrhizobium diazoefficiens* USDA110 after 10d (A), *Bacillus subtilis* NCIB3610 after 24h (B), *Burkholderia ambifaria* after 48h (C), *Herbaspirillum seropedicae* ATCC 33892 after 48h (D), *Paenibacillus polymyxa* after 72h (E), *Pseudomonas chlororaphis* after 48h (F), *Pseudomonas soyae* after 48h (G), and *Variovorax boronicumulans* after 48h (H).

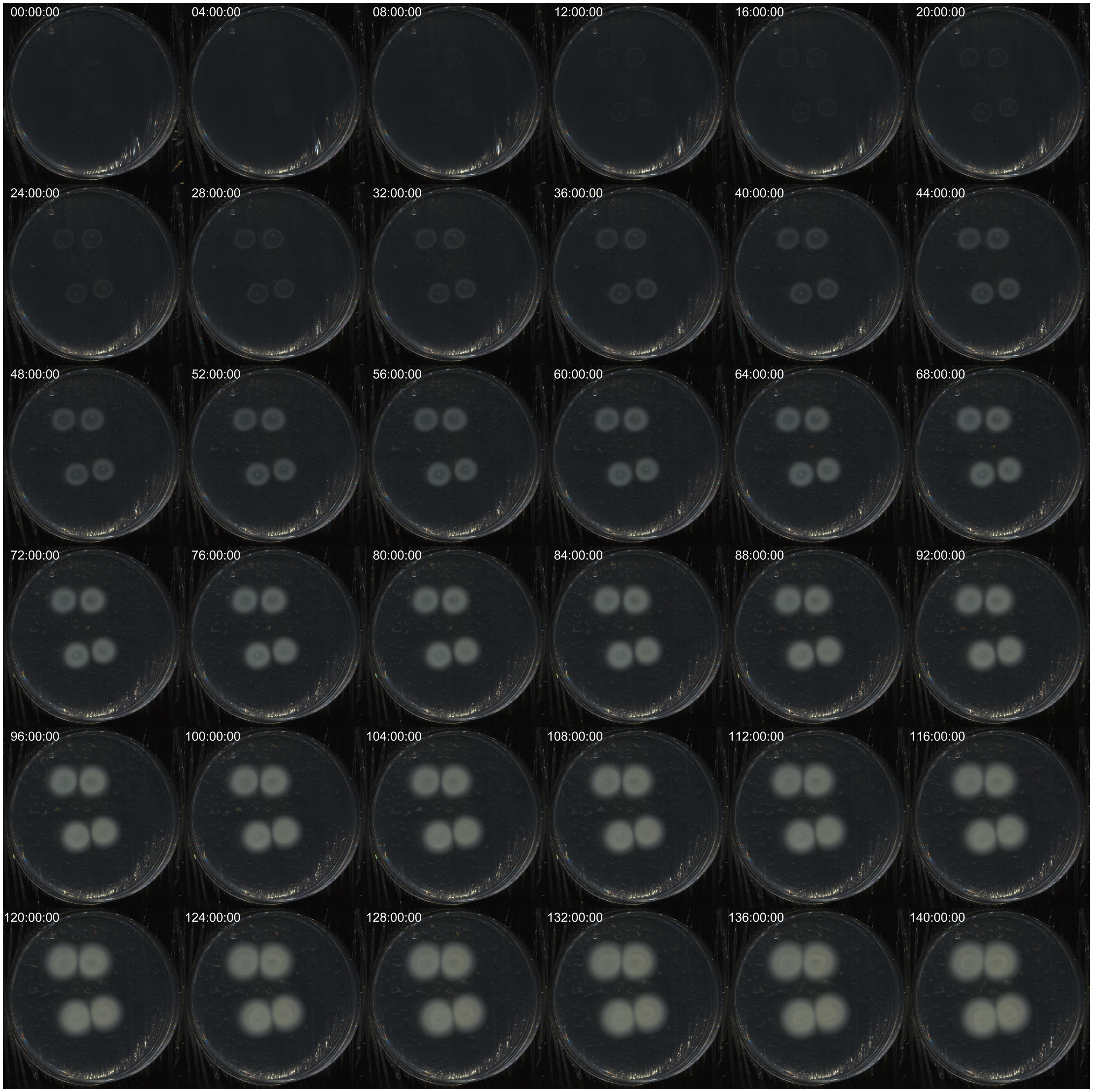

Figure S3 Four colony setup of *B. diazoefficiens* USDA110 in soft agar, imaged every 4h from d0 to d6

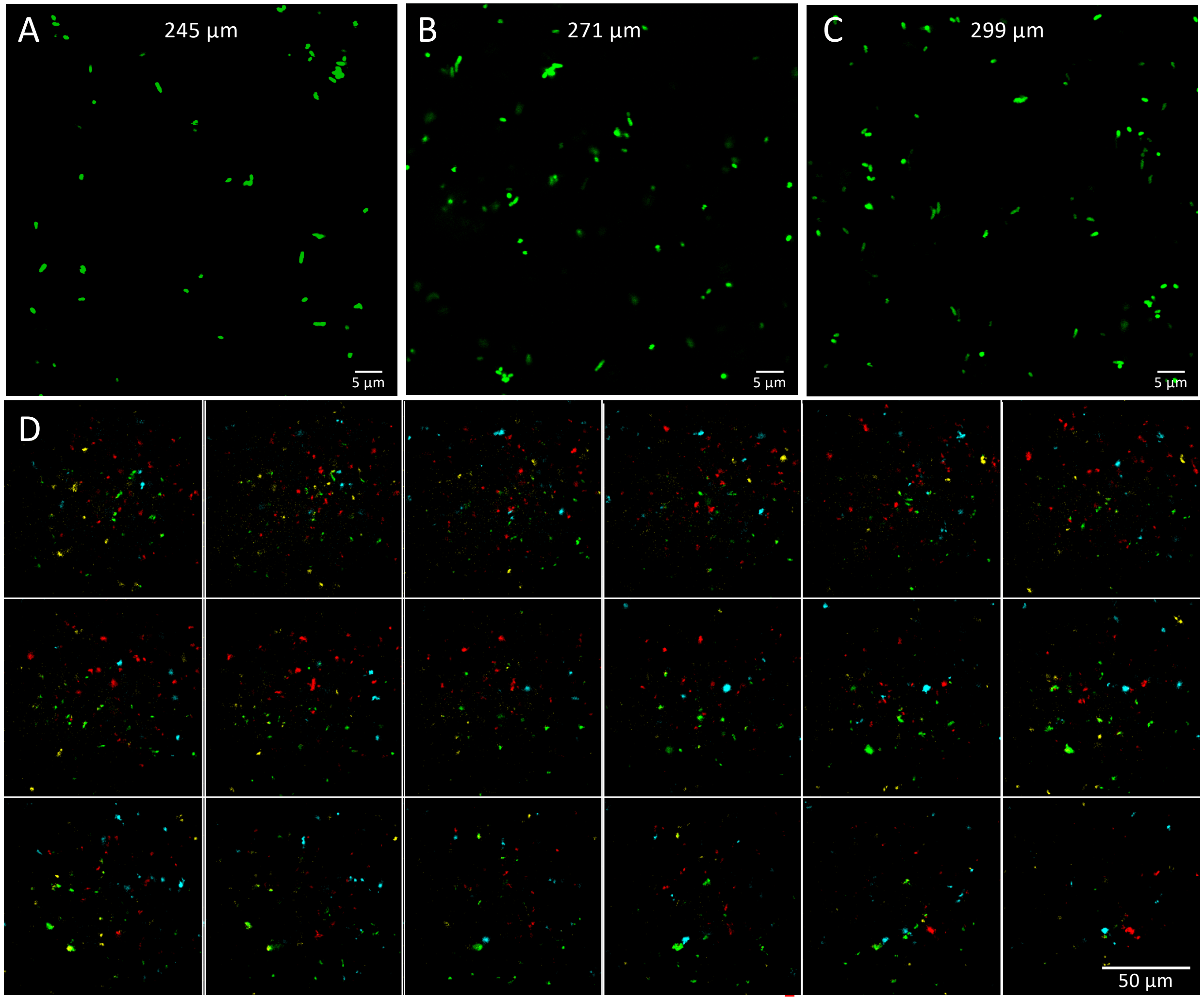

Figure S4. Distribution of USDA110 in soft agar viewed by confocal microscopy. Panels A - C show the cell distribution of bjGFP-labeled USDA 110 at 245, 271 and 299 µm. Panel D shows the distribution of four USDA 110 clones tagged with bjGFP, mChe, sYFP2 and mTq2, precultured separately and mixed in equal proportions before inoculation.

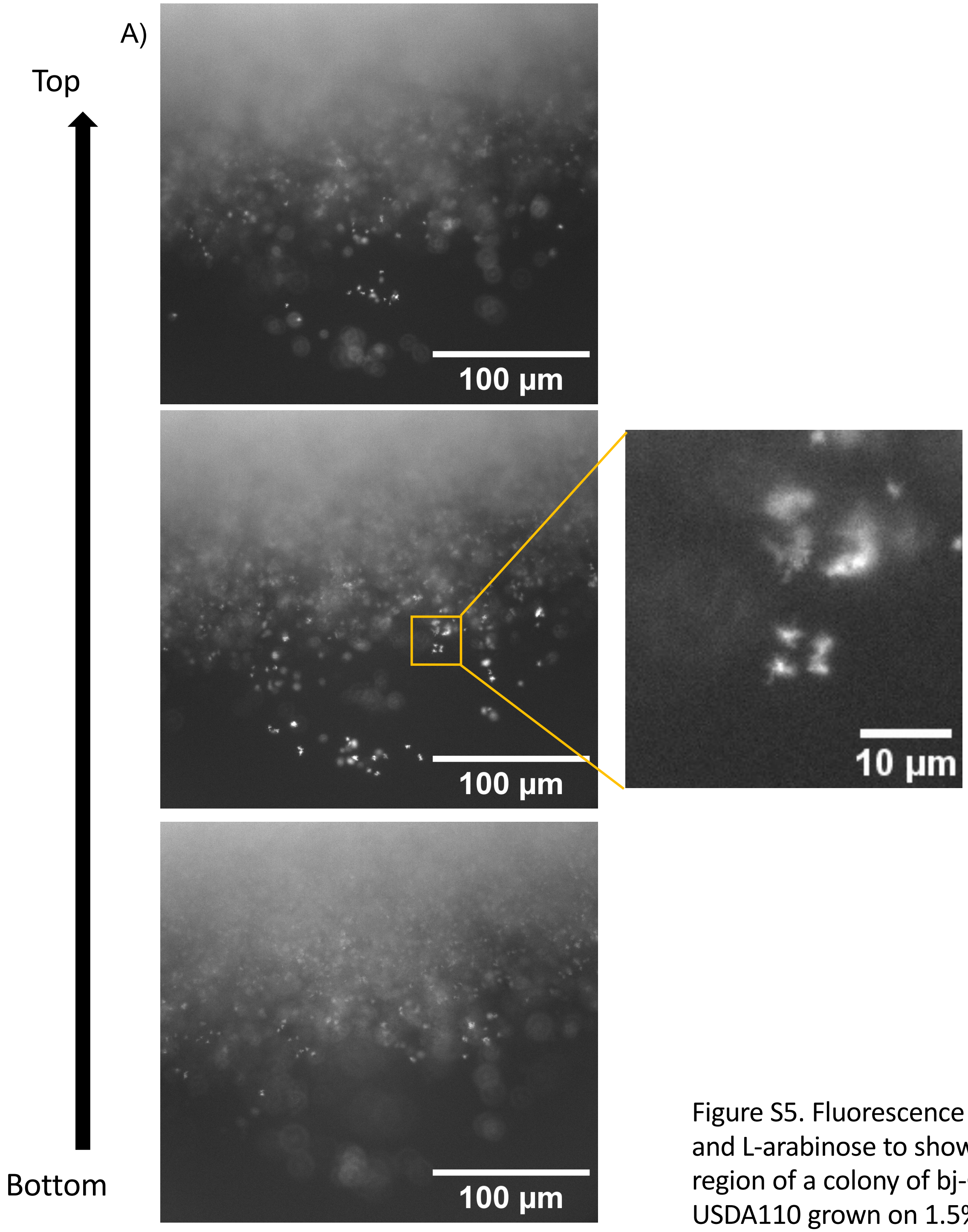

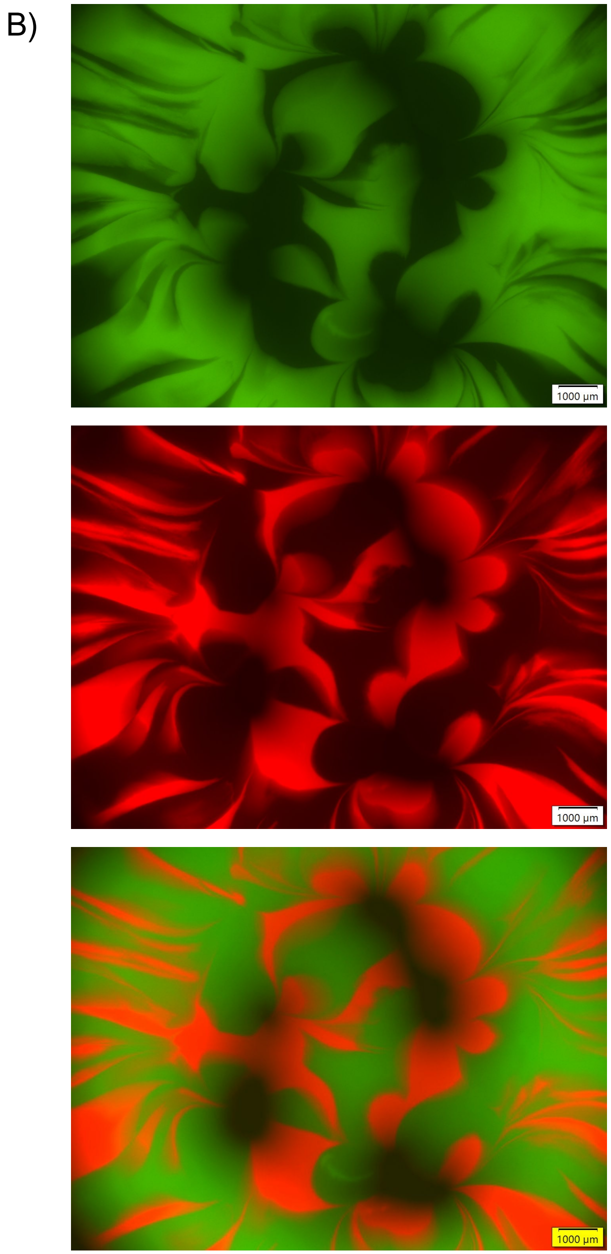

Figure S5. Fluorescence microcopy of *E. coli* (pGlo) swimming in LB with 0.35% agar and L-arabinose to show clustering among cells (A). Fluorescence micrograph of region of a colony of bj-GFP and mCherry-tagged strains of *B. diazoefficiencs* USDA110 grown on 1.5% agar (B).

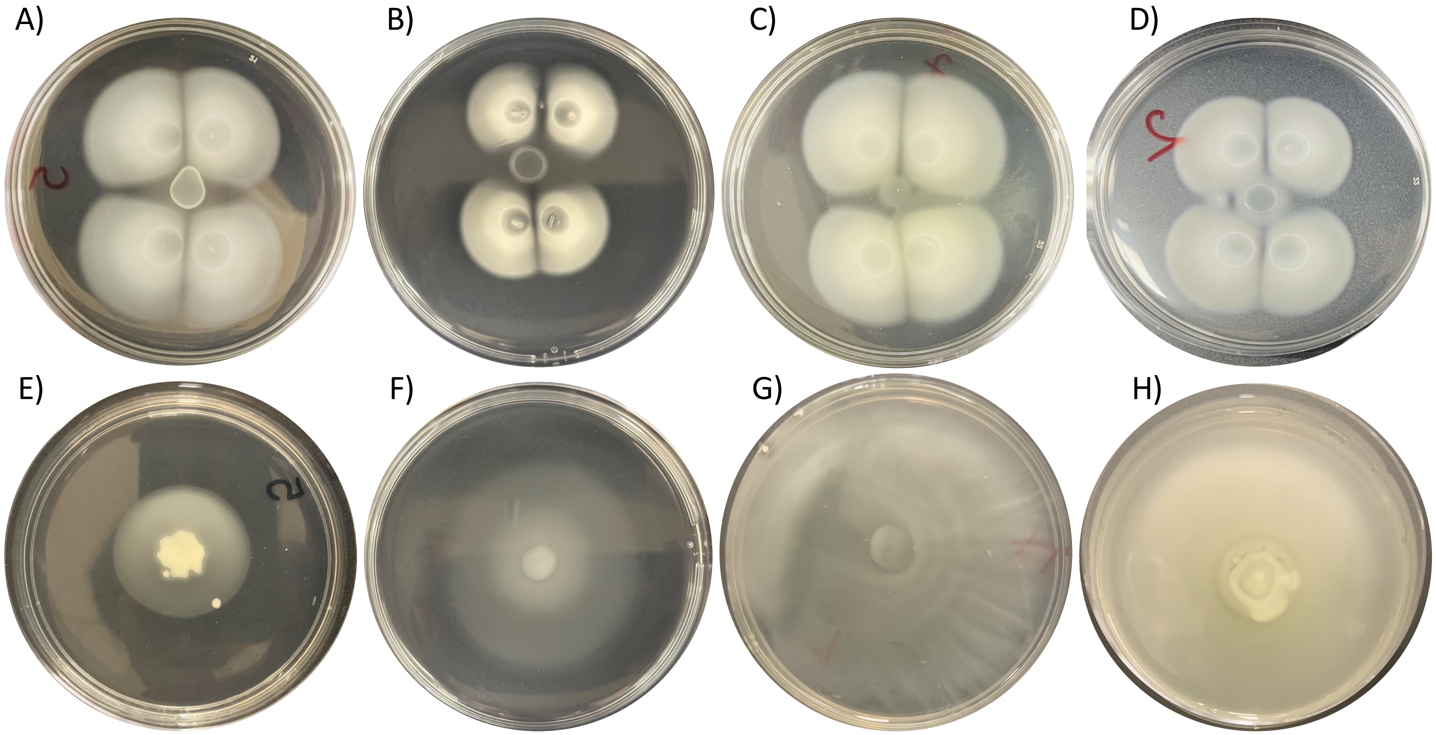

Figure S6 Inhibition of growth in the ICZ of swimming colonies of *B. diazoefficiens* USDA 110. Panels A - D were spot-inoculated onto the ICZ with *Arthrobacter aurescens* (A), *E. coli* (B), *Pseudomonas* ADP (C) and *Salmonella* Typhimurium (D), while panels E - H contained only the respective single cultures.

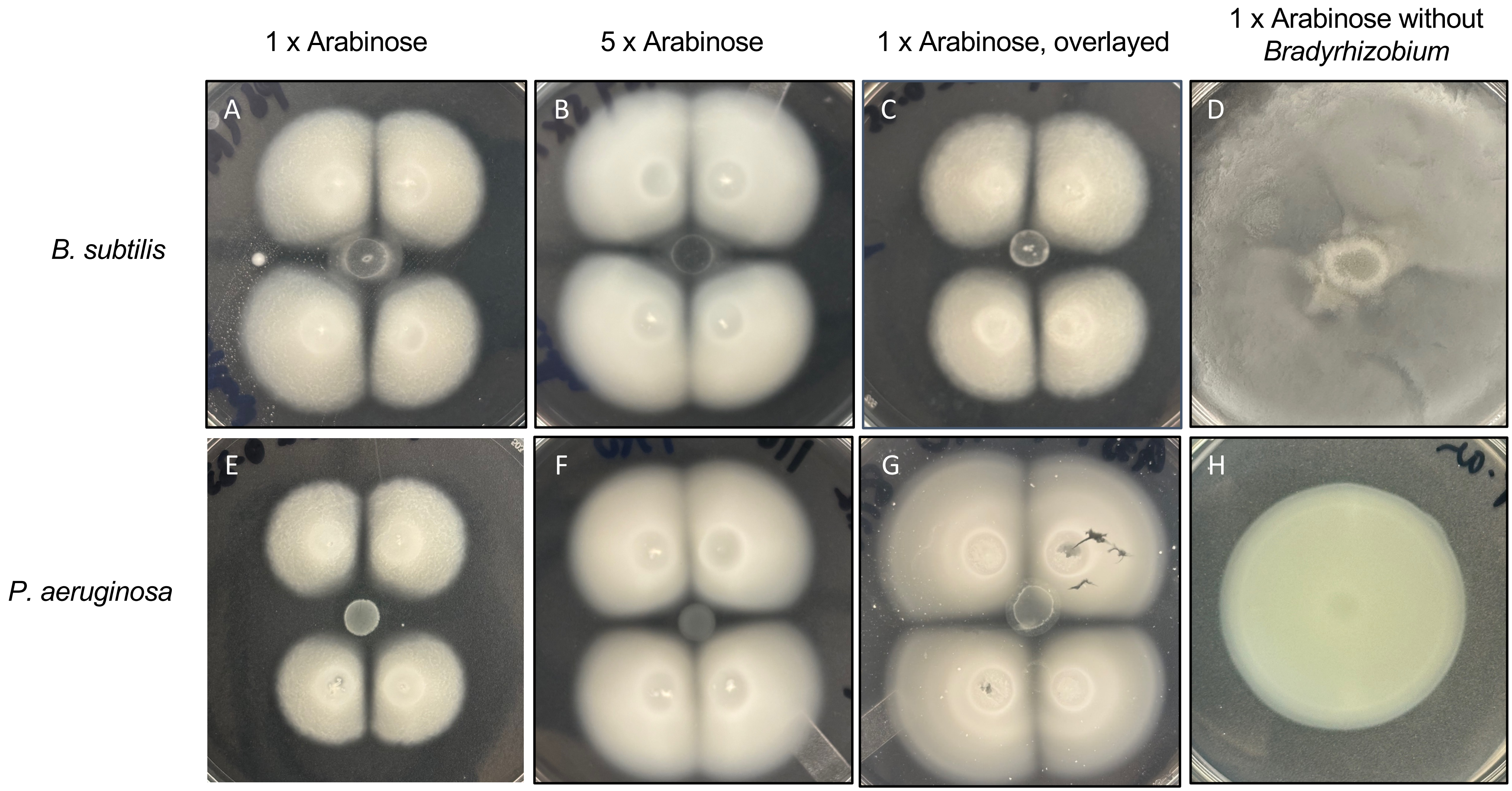

Figure S7. Effect of nutrient concentration on Inhibition of growth of *B. subtilis* (A – D) and *P. aeruginosa* (E – H). Exponentially growing cultures of *B. subtilis* and *P. aeruginosa* were spot-inoculated onto the ICZ of USDA 110 grown for 7 d in PSY with arabinose (A, E), PSY with 5x arabinose (B, F), PSY with 1x arabinose and then overlaid with 5x arabinose soft agar before spot inoculation (C, G), and *B. subtilis* and *P. aeruginosa* spot inoculated onto sterile PSY with arabinose (D, H).

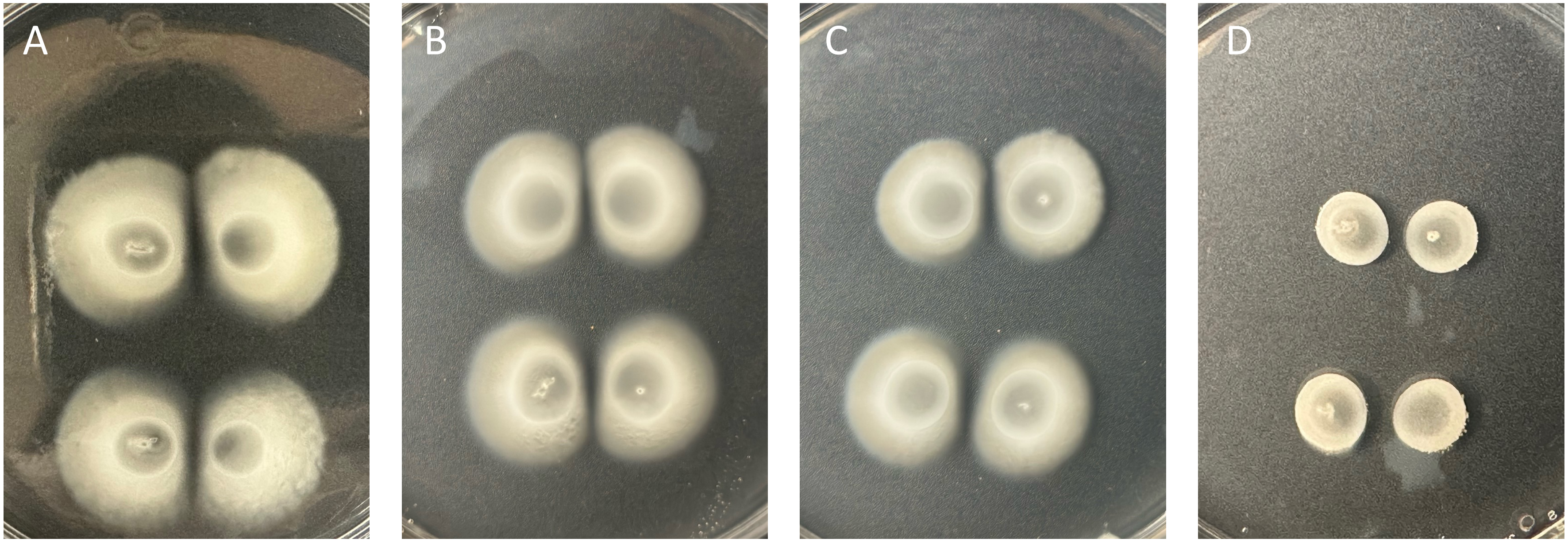

Figure S8 Four colony set-up of *Bradyrhizobium* USDA110-Spc4 (A), and its *fliC* (B)*, lafA* (C) and *fliC-lafA* (D) deletion mutants, incubated in PSY soft agar with arabinose for 7 d.

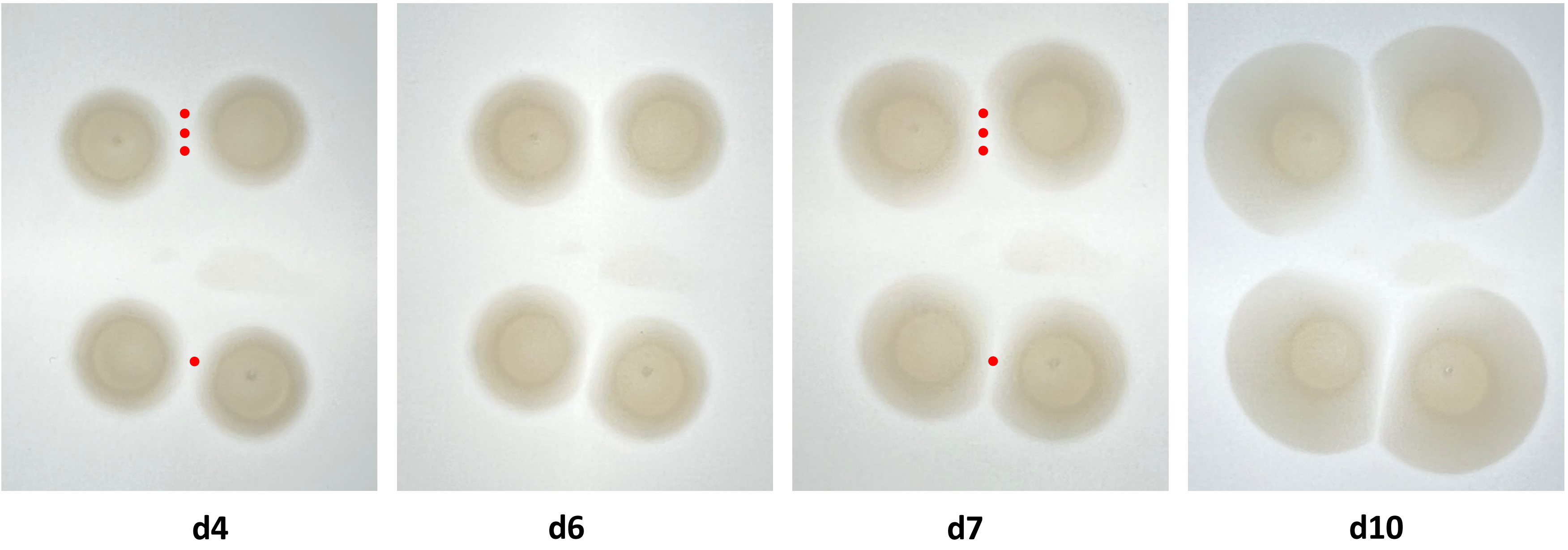

Figure S9 Four colony set-up of *Bradyrhizobium* USDA110 in PSY soft agar with arabinose, with 5µL of 10X PSY added on d 4 and again d7 at the positions marked by red dots.

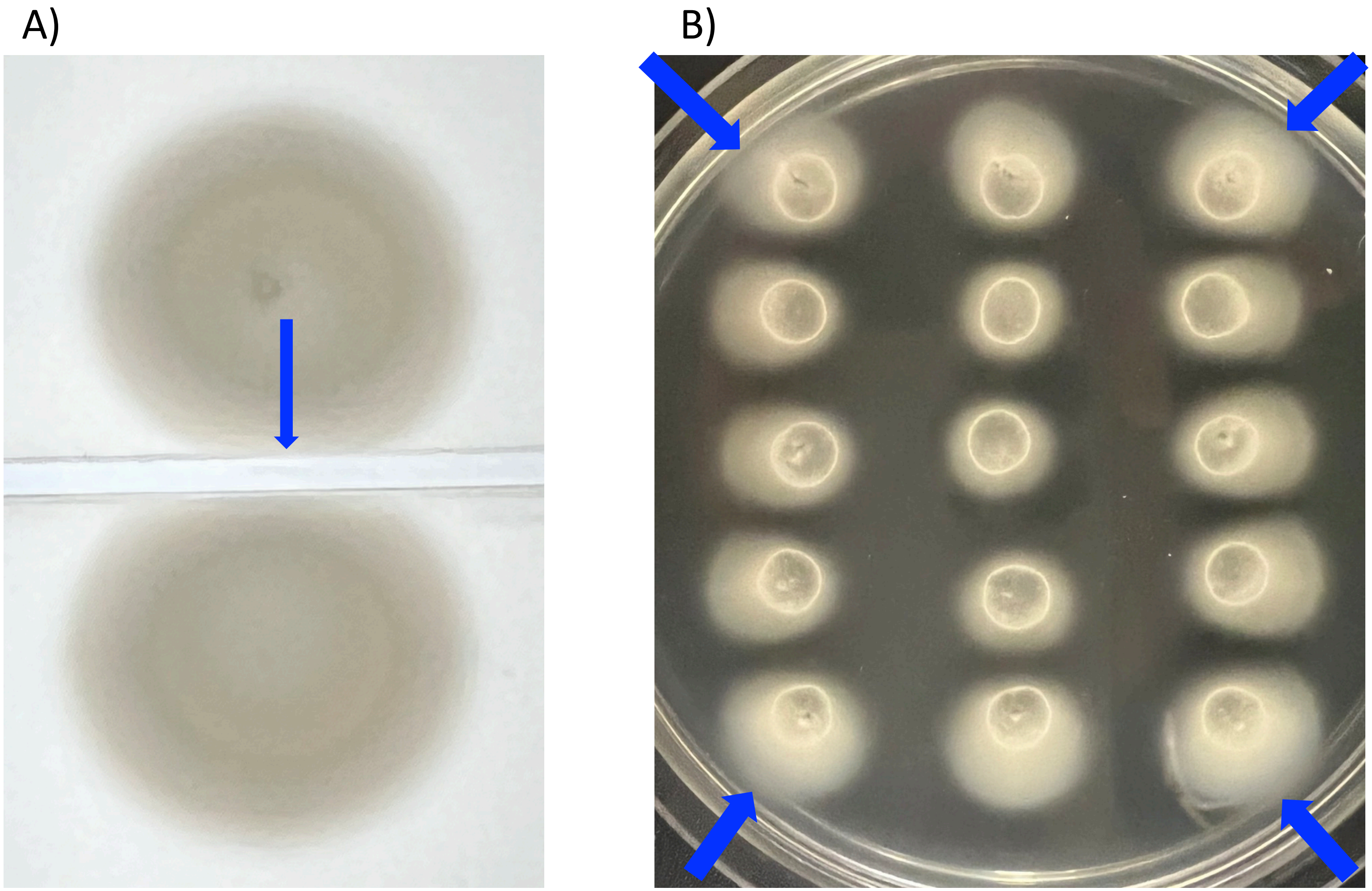

Figure S10 Swimming colonies of *Bradyrhizobium* USDA110 in PSY soft agar with arabinose, with a sterile glass slide placed between them (Aa), and colonies spotted close to the edge of a Petri dish (B).

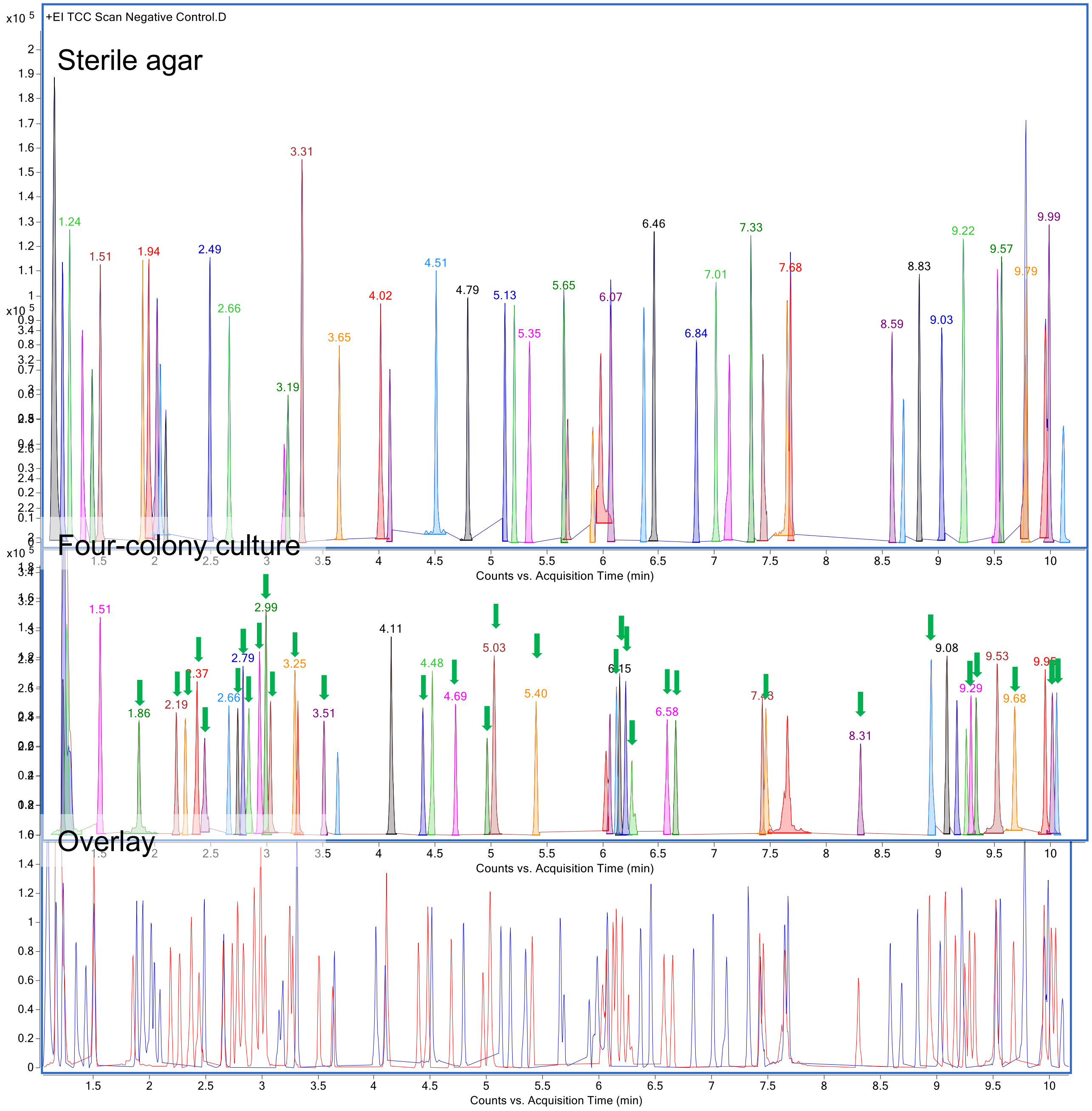

Figure S11 GC-MS traces of water left in transwell plates (0.4 µm pore size) on top of either sterile agar (top panel) or four-colony culture of *Bradyrhizobium* for 24h. Peaks unique to the four-colony setup are highlighted by green arrows. The last panel shows an overlay of the two traces (red = *Bradyrhizobium* and blue = sterile control).
