# Supplementary material for: Self-growth suppression in Bradyrhizobium diazoefficiens is caused by a diffusible antagonist
Source: ISME Commun. 2025 Feb 17;5(1):ycaf032. doi: 10.1093/ismeco/ycaf032 (PMC11896636; doi:10.1093/ismeco/ycaf032)
Supplement: Table_S1_ycaf032 [file table_s1_ycaf032.docx]

Table S1 Strains and plasmids used in this study

| **Bacterial strain** | **Source** | **Reference** |
| --- | --- | --- |
| *Arthrobacter aurescens* TC1 | Dr. Mike Sadowsky | [1] |
| *Bacillus subtilis* 168 | BGSC* |  |
| *Bacillus subtilis* NCIB 3610 | BGSC |  |
| *Bradyrhizobium arachidis* USDA 3384 | NRRL ** |  |
| *B. diazoefficiens* USDA 110 | NRRL |  |
| *B. diazoefficiens* USDA 110 *spc4* | Dr. Caroline Harwood | [2] |
| *B. diazoefficiens* USDA 110 spc4 *fliC,* devoid of subpolar flagellar filaments | Dr. Aníbal R. Lodeiro | [3] |
| *B. diazoefficiens* USDA 110 spc4 *laf A* and *fliC,* lacking both flagellar systems | Dr. Aníbal R. Lodeiro | [3] |
| *B. diazoefficiens* USDA 110 spc4 *lafA,* devoid of lateral flagellar filaments | Dr. Aníbal R. Lodeiro | [3] |
| *B. elkanii* USDA 26 | NRRL |  |
| *B. elkanii* USDA 83 | NRRL |  |
| *B. japonicum**** USDA 110 spc4 *bjaI* mutant (AL17) | Dr. Caroline Harwood | [2] |
| *B. japonicum* USDA 126 | NRRL |  |
| *B.* *japonicum* USDA 140 | NRRL |  |
| *B. japonicum* USDA 20 | NRRL |  |
| *B. japonicum* USDA 6 | NRRL |  |
| *B. liaoningense* USDA 13 | NRRL |  |
| *Burkholderia ambifaria* | Lab collection**** |  |
| *Escherichia coli* K12 | ATCC***** |  |
| *E. coli* S17-1 λpir | Nova Lifetech Inc |  |
| *Herbaspirillum seropedicae* ATCC 33892 | ATCC |  |
| *Paenibacillus polymyxa* | Lab collection |  |
| *Pseudomonas ADP* | Dr. Mike Sadowsky | [4] |
| *P. aeruginosa* PA0 | ATCC |  |
| *P. chlororaphis* | Lab collection |  |
| *P. soyae* | Lab collection |  |
| *Salmonella* Typhimurium | Lab collection |  |
| *Streptomyces* ATCC 49182 | ATCC |  |
| *Variovorax boronicumulans* | Lab collection |  |
|  |  |  |
| Plasmids used |  |  |
| pRJPaph-bjGFP-1 | Dr. Hans-Martin Fischer | [5] |
| pRJPaph-mTq2-1 | Dr. Hans-Martin Fischer | [5] |
| pRJPaph-sYFP2-1 | Dr. Hans-Martin Fischer | [5] |
| pRJPaph-mChe-1 | Dr. Hans-Martin Fischer | [5] |

* Bacillus Genetic stock Center

**NRRL Culture Collection of the Agricultural Research Service, USDA

*****  Till 2013 *B. diazoefficiens* USDA 110 was known as *B. japonicum* USDA 110 [6]

**** Isolates in the lab collection were obtained from soils, and their species names derived from closest aligning species at NCBI based on V1-9 region of their 16SrRNA genes.

***** American Type Culture Collection

[1] L.C. Strong, C. Rosendahl, G. Johnson, M.J. Sadowsky, and L.P. Wackett, *Arthrobacter aurescens* TC1 metabolizes diverse s-triazine ring compounds. Applied and Environmental Microbiology 68 (2002) 5973-5980.

[2] A. Lindemann, G. Pessi, A.L. Schaefer, M.E. Mattmann, Q.H. Christensen, A. Kessler, H. Hennecke, H.E. Blackwell, E.P. Greenberg, and C.S. Harwood, Isovaleryl-homoserine lactone, an unusual branched-chain quorum-sensing signal from the soybean symbiont *Bradyrhizobium japonicum*. Proceedings of the National Academy of Sciences 108 (2011) 16765-16770.

[3] F. Mengucci, C. Dardis, E.J. Mongiardini, M.J. Althabegoiti, J.D. Partridge, S. Kojima, M. Homma, J.I. Quelas, and A.R. Lodeiro, Characterization of FliL proteins in *Bradyrhizobium diazoefficiens*: lateral FliL supports swimming motility, and subpolar FliL modulates the lateral flagellar system. Journal of bacteriology 202 (2020) e00708-19.

[4] M. De Souza, L.P. Wackett, K.L. Boundy-Mills, R.T. Mandelbaum, and M.J. Sadowsky, Cloning, characterization, and expression of a gene region from *Pseudomonas* sp. strain ADP involved in the dechlorination of atrazine. Applied and environmental microbiology 61 (1995) 3373-3378.

[5] R. Ledermann, I. Bartsch, M.N. Remus-Emsermann, J.A. Vorholt, and H.-M. Fischer, Stable fluorescent and enzymatic tagging of *Bradyrhizobium diazoefficiens* to analyze host-plant infection and colonization. Molecular Plant-Microbe Interactions 28 (2015) 959-967.

[6] J.R.M. Delamuta, R.A. Ribeiro, E. Ormeno-Orrillo, I.S. Melo, E. Martínez-Romero, and M. Hungria, Polyphasic evidence supporting the reclassification of *Bradyrhizobium japonicum* group Ia strains as *Bradyrhizobium diazoefficiens* sp. nov. International Journal of systematic and evolutionary microbiology 63 (2013) 3342-3351.
